# Examination of the influence of cedar fragrance on cognitive function and behavioral and psychological symptoms of dementia in Alzheimer type dementia

**DOI:** 10.1002/npr2.12096

**Published:** 2020-02-09

**Authors:** Yuya Takahashi, Sho Shindo, Takashi Kanbayashi, Masahiro Takeshima, Aya Imanishi, Kazuo Mishima

**Affiliations:** ^1^ Department of Neuropsychiatry Akita University Graduate School of Medicine Akita Japan; ^2^ Akita Research Institute of Food and Brewing Akita Japan; ^3^ International Institute for Integrative Sleep Medicine (WPI‐IIIS) University of Tsukuba Tsukuba Japan

**Keywords:** alzheimer type dementia, aroma extracts from ceder, cognitive disorders, cognitive neuoscience, dementia, olfactory nerve

## Abstract

We examined whether symptoms of dementia are improved by olfactory nerve stimulation in Alzheimer type dementia patients. First, a stick‐type olfactory identification ability test was performed in patients with Alzheimer type dementia, to select patients without olfactory dysfunctions. Then, these patients were randomly assigned into the intervention (n = 19) and the control groups (n = 17). To evaluate the effects of olfactory nerve stimulation, we exposed the intervention group to a disinfecting ethanol with added aroma extracts from ceder and the control group to the ethanol without the added aroma extracts. Each group underwent the intervention for 8 weeks, cognitive and behavioral functions were evaluated before and after treatments using the Neuropsychiatric Inventory (NPI), the Japanese version of Zarit Caregiver Burden interview (J‐ZBI), and the Alzheimer's Disease Assessment Scale‐cognitive subscale (ADAS‐cog). A significant improvement was observed in the NPI score and J‐ZBI in the intervention group compared to the control group at 4 and 8 weeks. On the other hand, there was no significant difference in the score of ADAS‐cog. Exposure to cedar fragrance improved behavioral and psychological symptoms of dementia (BPSD) in Alzheimer type dementia and may reduce the burden of nursing care. In addition to its effectiveness, the procedure is simple and minimally invasive and would be a valuable non‐pharmaceutical treatment.

**Abbreviations**
AD: Alzheimer type dementia
NPI: Neuropsychiatric Inventory
J‐ZBI: The Japanese version of the Zarit Caregiver Burden interview
ADAS‐cog: Alzheimer 's Disease Assessment Scale ‐ cognitive subscale
BPSD: Behavioral and Psychological Symptoms of Dementia

## 1 INTRODUCTION

Treatment options for Alzheimer type dementia (AD) are currently limited, and new treatment methods are awaited. Acetylcholine‐esterase inhibitors and a glutamate receptor inhibitor are the commonly used pharmacological treatments, however, their effects are limited. Side effects such as gastrointestinal symptoms, trembling and drowsiness are also problematic. As non‐pharmaceutical treatments, reminiscence therapy and reality orientation are known, but the effects are also not clear and patient compliance is necessary for these treatments. A study has reported an improvement in cognitive function by olfactory stimulation using aromatherapy^1^, and this may be a potential alternative non‐pharmaceutical option. Yet, reports on this treatment are limited in Japan, and the effectiveness has not been elucidated.

1. Treatment of dementia with olfactory stimulation.

In recent years, olfactory nerve transport has attracted attention as a method to bypass the blood brain barrier. In animal experiments, nasally administered insulin growth factor 1 (IGF‐1), is absorbed from the nasal mucosa, then migrates to the olfactory bulb, hippocampus, pons, and upper spinal cord.^2^ In clinical trials, insulin reaches the cranial nerve in about 30 minutes after intranasal administration. Nasal administration of insulin was also shown to be effective for improving cognitive function in those with AD and mild cognitive impairment.^3^ The olfactory stimulus spread through the olfactory nerve, is transmitted to the limbic system and the hypothalamus. The limbic system is a region that includes the hippocampus and amygdala, which are closely related to cognitive impairment. Thus olfactory stimulation may cause improvement of cognitive impairment. Furthermore, stimulation to the hypothalamus accelerates the autonomic nervous system and can also be expected to improve psychiatric symptoms such as anxiety and depression. Fragrance exposure can be carried out comparatively easily, and the burden on the subject is small.

2. An ethanol for environmental disinfection has been developed from Akita cedar using biorefinery technology.^4^

This bioethanol has cedar‐derived antibacterial activity and fragrance. The antibacterial activity has been confirmed to be equivalent to that of conventional disinfecting ethanol. The fragrance of Akita cedar is familiar and well‐liked among the elderly people in Japan, and a healing effect could also be expected. Traditionally, the cedar fragrance has been believed to have various health effects, but few reports have directly examined its effects on cognitive function.

On the other hand, olfactory sensation weakens with age as well as other sensations such as vision and hearing. Furthermore, olfactory function is known to be impaired in neurodegenerative diseases, such as AD, from the early stage.^5^ Many problems such as inability to practice fire safety and cleanliness, dysgeusia, and loss of appetite occur due to olfactory dysfunction.^6^ These dysfunctions have large influences on the daily life of dementia patients. A correlation between the olfactory function and the brain region involved in cognitive function and memory has been reported.^7^ A correlation between the function of olfactory identification and the volume of the hippocampus/parahippocampal region of the MRI image has also been reported.^8^ For this reason, olfactory dysfunctions have attracted attention as a biomarker for early diagnosis of dementia and prediction of onset.

As described above, cognitive function, behavioral, and psychological symptoms of dementia (BPSD), and olfactory functions are closely related to each other. Olfactory stimulation may lead to a new non‐invasive treatment for dementia. However, because olfactory function may be declined in patients with dementia, the ability to transport substances in the olfactory nerve to brain is reported to be decreased.^9^

In this study, we first confirm the presence or absence of olfactory dysfunction by using the “Odor Stick Identification Test for Japan (OSIT‐J)” on AD patients. We then examined whether symptoms of dementia are improved by olfactory stimulation in the AD patients without olfactory impairment. For the olfactory stimulation, ethanol for environmental disinfection with added cedar fragrance was used.

## 2 PATIENTS AND METHODS

### 2.1 Methods of aroma exposure

**Figure 1:**
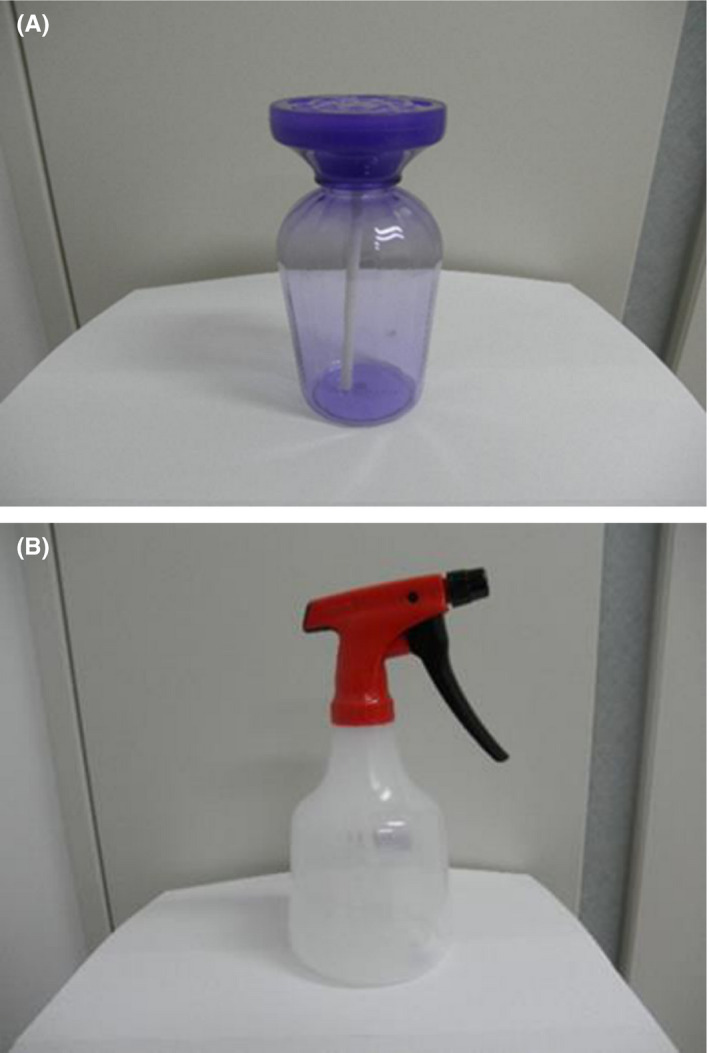
A, Room Fragrance Type. B, Spray Type

Twenty grams of cedar leaves were cut into 1cm strips and added to 200 mL of a 20% ethanol solution. This solutions was distilled to 50% ethanol at 60°C under a lowered pressure (170 hpa) with a rotary evaporator. This product is a new environment disinfecting ethanol that has antibacterial ability and an added fragrance derived from Akita Cedar leaves. From each environmental disinfectant ethanol (with and without added cedar fragrance), room fragrance type and spray type dispensers were prepared. The room fragrance type (Figure 1A) can diffuse the fragrance ingredients using rattan sticks, and 2.3 mL of distilled liquid is delivered per day at room temperature. The spray type (Figure 1B) is used a few times a day. The room fragrance type is placed in the resident space (living room and bedroom), and the spray type is used to mist the patients’ clothing and bedding.

### 2.2 Subjects and methods

We diagnosed AD using the AD clinical diagnostic standard by NINCDS/ADRDA. We treated probable AD as AD and did not include those with mild cognitive impairment (MCI). For outpatient AD subjects, we conducted the stick‐type olfactory identification test method (OSIT‐J) and selected patients who answered more than 9 out of 12 items correctly, as they were recognized as not having olfactory dysfunctions. These patients were assigned randomly to the intervention group or the control group. The intervention group consisted of 19 persons (12 female and 7 male), and the mean age ± standard deviation (SD) was 76.2 ± 9.8. The control group had 17 persons (10 female and 7 male) and had a mean age ± SD of 75.8 ± 7.8. There was no difference between age and sex in each group. The score of OSIT‐J in the intervention group was 10 ± 1.1 and in the control group was 11 ± 1.2.

After a 4 week pretreatment period, both groups were exposed to the environment disinfecting ethanol for 8 weeks. The ethanol with added cedar fragrance was used for the intervention group and the ethanol without added cedar fragrance was used for the control group. In order to evaluate the effect of aroma exposure on cognitive function, each group was evaluated at the beginning of exposure and at 4 and 8 weeks. We implemented the Neuropsychiatric Inventory (NPI), the Japanese version of the Zarit Caregiver Burden interview (J‐ZBI)^10^ to assess BPSD, and the Alzheimer's Disease Assessment Scale‐cognitive subscale (ADAS‐cog) to assess cognitive function. In both the intervention and the control groups, the pharmaceutical treatments for dementia still continued without changes from the start of the exposure to the end.

### 2.3 Statistical analysis

For statistical analysis, gender, age, history of morbidity, educational years, and Functional Assessment Staging (FAST) in the intervention and control groups were compared using the Mann‐Whitney's U test. In addition, at 4 and 8 weeks after the start of exposure, the NPI, J‐ZBI, and the ADAS‐cog scores were compared between the 2 groups using the Student's t test. The significance level was set to 5% (*P* < .05).

## 3 RESULTS

Table 1 shows the demographic information of the intervention group (ethanol with added cedar fragrance) and the control group (ethanol without added fragrance). There was no significant difference in sex, age at the time of examination, education years, each item of the FAST and OSIT‐J scores between the intervention and the control groups. Most patients with dementia were in the mild to moderate stages in FAST. There were 15 out of 19 patients in the intervention group and 14 out of 17 patients in the control group who used anti‐dementia medications; there was no significant difference in the proportion of patients who used anti‐dementia medication and of patients who did not use the medication.

**Table 1:** Background of Intervention and control groups. There was no significant difference between the 2 groups in back ground

	Intervention group (N = 19)	Control group (N = 17)
Female, n(%)	12 (63.2)	10 (58.8)
Age, mean(SD), yr	76.2 (9.8)	75.8 (7.8)
Duration of illness, mean(SD), yr	2.2 (1.5)	2.1 (0.9)
Education, mean(SD), yr	9.7 (1.6)	9.6 (2.4)
FAST, mean(SD)	2.6 (1.1)	3.0 (0.9)
People on dementia medications, n(%)	15 (78.9)	14 (82.4)
OSIT‐J score, mean(SD)	10 (1.1)	11 (1.2)

Abbreviations: FAST, Functional Assessment Staging; OSIT‐J, the Odor Stick Identification Test for Japan; SD, standard deviation.

**Figure 2:**
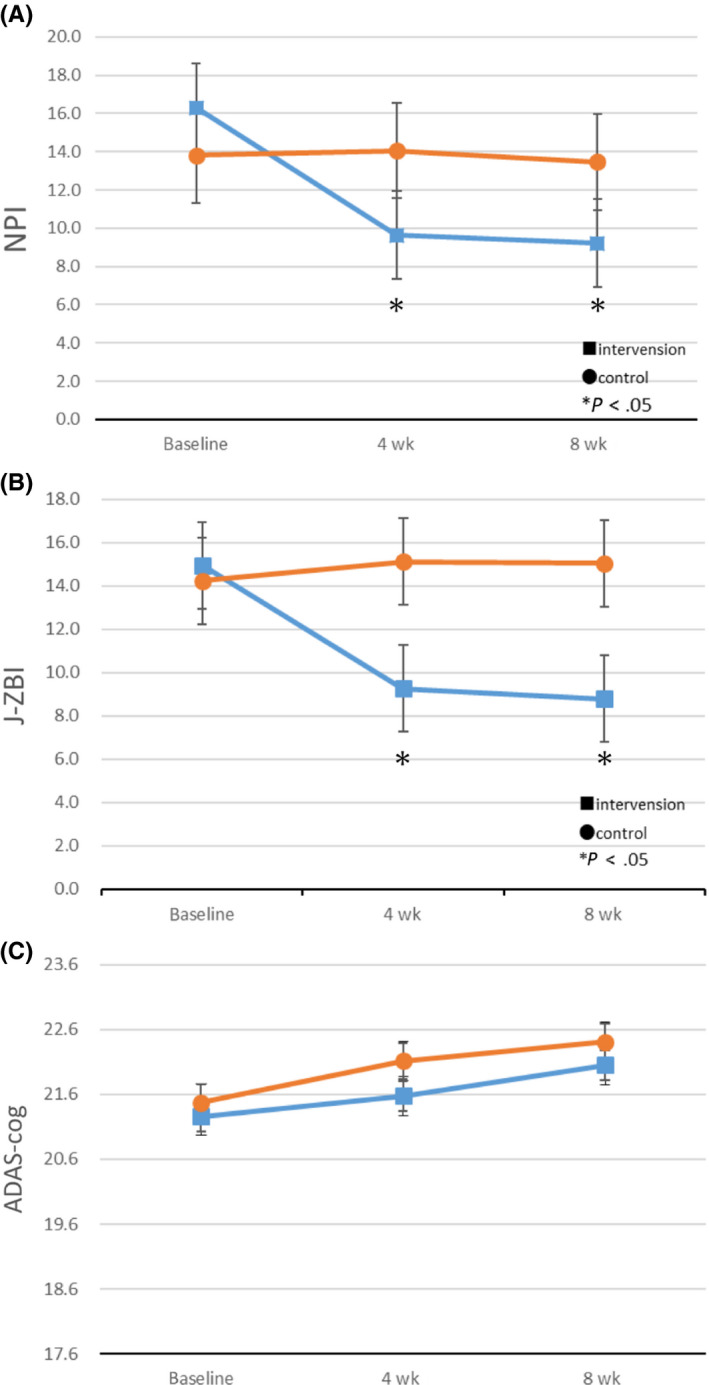
A, In the intervention group, the NPI score improved significantly after 4 and 8 wks. B, In the intervention group, the Japanese version of the Zarit Caregiver Burden interview (J‐ZBI) significantly decreased after 4 and 8 wks. C, There was no significant difference in ADAS ‐ cog between the intervention and the control groups

Figure 2 compares the results of NPI, J‐ZBI, and ADAS‐cog scores, at the start of exposure and at 4 and 8 weeks for the intervention and control groups. For NPI, the score of the intervention group significantly decreased compared to the control group at 4 weeks and 8 weeks (Figure 2A). Similarly, the score of the J‐ZBI was significantly lower in the intervention group than in the control group at 4 and 8 weeks (Figure 2B).

**Figure 3:**
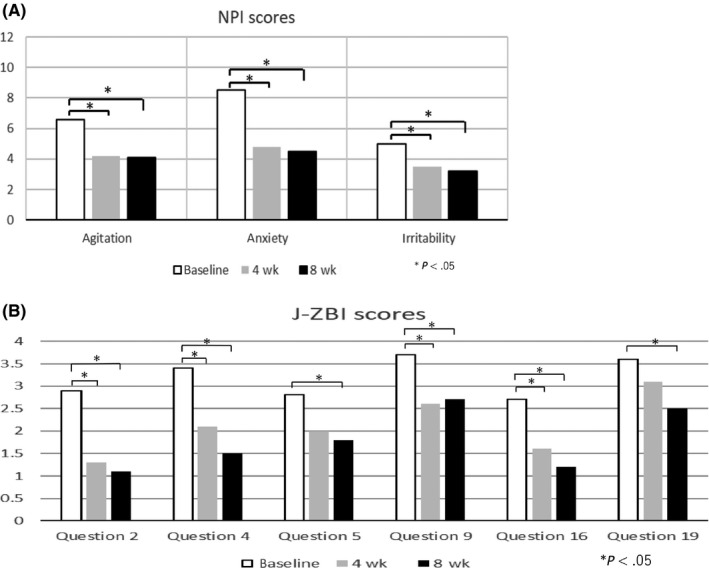
A, This figure shows the following 3 out of 10 items of NPI, in which significant differences were observed: agitation, anxiety, and irritability. B, This figure shows the following 6 out of 22 items of J‐ZBI, in which significantly differences were observed: (Q2) Not having enough time for yourself, (Q4) Embarrassed of patient behavior, (Q5) Feel angry around patient, (Q9) Feel strained around patient, (Q16) Feel unable to take care of the patient much and (Q19) Feel uncertain of what to do

Therefore, the results suggest that exposure to cedar fragrance may improve BPSD of AD patients, which may reduce the care burden of caregivers. There was no significant difference between the intervention group and the control group in ADAS‐cog score (Figure 2C). Figure 3‐a shows the following 3 out of 10 items of NPI, in which significant differences were observed: agitation, anxiety, and irritability. Figure 3B shows the following 6 out of 22 items of J‐ZBI, in which significantly differences were observed: (Q2) Not having enough time for yourself, (Q4) Embarrassed of patient behavior, (Q5) Feel angry around patient, (Q9) Feel strained around patient, (Q16) Feel unable to take care of the patient much and (Q19) Feel uncertain of what to do.

## 4 DISCUSSION

There are few previous studies on the effect on aromatherapy for symptoms of dementia. Fuzii et al reported that lavender aroma therapy is effective for BPSD,^11^ and their results are consistent with our results. The method of stimulating the olfactory nerve by fragrance exposure is easy to implement because of its low invasiveness and can be expected to improve the BPSD. However, this approach must consider that similar stimulus reaches not only to patients but also to caregivers. BPSD of patients is reported to improve when the mental state and attitude of caregivers is good.^12^, ^13^ Even in this study, there is a possibility that the reported BPSD of patients and perceived care burden degree were alleviated because of aroma exposure to the caregivers.

Out of the 10 domains of the NPI, improvement was observed in 3 domains such as, agitation, anxiety and irritability. Six out of 22 items in the J‐ZBI showed significant improvement, and these indicators are classified into dimensions of consequences of caregiving (Q2, 5, 9) and exhaustion and uncertainty (Q4, 16, 19)^14^. The relaxation effect of the cedar fragrance affects both the AD patients and the caregivers and may contribute to the improvement of BPSD and reduction of care burden.

There is a report that the cognitive function itself is improved by olfactory stimulation,^15^ but our results did not show improvement of ADAS‐cog, which is an indicator of cognitive function. It is difficult to clarify whether the difference is due to the fragrance component or the stimulation method. Since we chose a group of patients without olfactory dysfunction in this experiment, the results may be influenced from selecting patients who have do not have advanced pathology of degeneration.

Olfactory impairment is often observed in AD patients in advance of cognitive decline.^16^ It is also reported that cognitive function tends to decline if there is olfactory dysfunction.^17^ Risks of AD^18^ and Parkinson's disease^19^ are increased with existences of olfactory dysfunction. Therefore, patients with olfactory dysfunction are closely associated to the onset of dementia. It may be possible that another degenerative pathology exists in the group of dementia patients without olfactory dysfunction. Yet, the changes seen in NPI score and J‐ZBI by olfactory stimulation were meaningful indications for future treatments for dementia.

Olfactory stimulation has very minimal invasiveness and is a highly feasible procedure. Conventionally, a comfortable environment has been known to suppresses the progress of AD,^20^, ^21^, ^22^ and our results could be explained due to the pleasing fragrance improving the environment. Aromatherapy would be a promising option in the treatment of dementia including AD. However, elderly people with dementia in Japan may have negative emotions against aromatherapy if they are unaccustomed to it. The current cedar scent is described as a fresh citrus sweet scent among professional perfume specialist. Since the cedar fragrance is familiar to the Japanese people, nostalgia, may be felt, and calming and healing effects could be achieved by exposure to it. In this study, no subjects complained of discomfort from the fragrance exposure and instead the fragrance was accepted favorably as a smell the subjects had experienced in the past. In Japan, the cedar fragrance maybe a useful olfactory stimulant for the elderly. It is expected to be a substitute for a disinfectant because it has the same sterilizing effect as a conventional disinfectant solution. Furthermore, the scent of cedar may give a healing effect and improves BPSD of dementia and relieves the care burden of caregiver.

### 4.1 Limitations

The majority of patients in this study received concurrent pharmaceutical treatments with anti‐dementia drugs. Although we did not change these treatments for dementia during the experimental period, there is a possibility that the use of anti‐dementia drugs may have influenced the NPI, J‐ZBI, and ADAS‐cog scores. Because we targeted patients who do not show olfactory dysfunction, it is not clear whether all dementia patients will have similar results. Further studies are needed to explore these issues.

## 5 CONCLUSION

In this study, we examined fragrance stimulation using cedar scent for AD patients without olfactory dysfunction. Compared to the control group, the intervention group significantly improved the NPI scores and J‐ZBI. Aroma exposure using cedar fragrance can improve BPSD in AD and can reduce the burden of nursing care. The procedure is simple and minimally invasive, so it was considered to be effective as a non‐pharmaceutical treatment.

## CONFLICT OF INTEREST

There is no conflict of interest of the authors with respect to this paper.

## AUTHOR CONTRIBUTIONS

All authors contributed toward data analysis and revision of the manuscript and agree to be accountable for all aspects of this study.

## APPROVAL OF THE RESEARCH PROTOCOL BY AN INSTITUTIONAL REVIEWER BOARD

This study was approved by the Akita University Ethics Committee.

## INFORMED CONSENT

An explanation concerning the study by the attending physicians were made, including consideration for anonymity so that individual patient could not be identified. Patients gave informed consent.

## Data Availability

All relevant data are included in Supporting Information.
